# Multidirectional hybrid algorithm for the split common fixed point problem and application to the split common null point problem

**DOI:** 10.1186/s40064-016-3691-2

**Published:** 2016-11-24

**Authors:** Xia Li, Meifang Guo, Yongfu Su

**Affiliations:** 1 Deprtment of Mathematics and Sciences, Hebei GEO University, Shijiazhuang, 050031 China; 2 Department of Mathematics, Tianjin Polytechnic University, Tianjin, 300387 China

**Keywords:** Split common fixed point problem, Split common null point problem, Fixed point, Metric resolvent, Multidirectional hybrid algorithm, Duality mapping, 47H05, 47H09, 47H10

## Abstract

In this article, a new multidirectional monotone hybrid iteration algorithm for finding a solution to the split common fixed point problem is presented for two countable families of quasi-nonexpansive mappings in Banach spaces. Strong convergence theorems are proved. The application of the result is to consider the split common null point problem of maximal monotone operators in Banach spaces. Strong convergence theorems for finding a solution of the split common null point problem are derived. This iteration algorithm can accelerate the convergence speed of iterative sequence. The results of this paper improve and extend the recent results of Takahashi and Yao (Fixed Point Theory Appl 2015:87, 2015) and many others .

## Introduction and preliminaries

Let $H_1$ and $H_2$ be two real Hilbert spaces, let $D\subset H_1$ and $Q\subset H_2$ be nonempty closed, and convex subsets, let $A : H_1\rightarrow H_2$ be a bounded linear operator. Then the split feasibility problem (Censor and Elfving 1994) is to find $z \in H_1$ such that $z \in D \cap A^{-1}Q$. Defining $U = A^*(I-P_Q)A$ in the split feasibility problem, we see that $U : H_1\rightarrow H_1$ is an inverse strongly monotone operator (Alsulami and Takahashi 2014), where $A^*$ is the adjoint operator of *A* and $P_Q$ is the metric projection of $H_2$ onto *Q*. Furthermore, if $D\cap A^{-1}Q$ is nonempty, then

1 $$\begin{aligned} z \in D \cap A^{-1}Q \ \Leftrightarrow \ z = P_D(I-\lambda A^*(I - P_Q)A)z, \end{aligned}$$

where $\lambda > 0$ and $P_D$ is the metric projection of $H_1$ onto *D*. Using such results regarding nonlinear operators and fixed points, many authors have studied the split feasibility problem in Hilbert spaces; see, for instance, Alsulami and Takahashi (2014), Byrne et al. (2012), Censor and Segal (2009), Moudafi (2010), Takahashi et al. (2015). Recently, Takahashi (2014) and Takahashi (2015) extended an equivalent relation as in (1) in Hilbert spaces to Banach spaces and then obtained strong convergence theorems for finding a solution of the split feasibility problem in Banach spaces. Very recently, using the hybrid method by Nakajo and Takahashi (2003) in mathematical programming, Alsulami et al. (2015) proved strong convergence theorems for finding a solution of the split feasibility problem in Banach spaces; see also Ohsawa and Takahashi (2003), Solodov and Svaiter (2000). Takahashi (2015) also obtained a result for finding a solution of the split feasibility problem in Banach space from the idea of the shrinking projection method by Takahashi et al. (2008). Takahashi and Yao (2015) presented the following hybrid iteration algorithm in a Hilbert space *H*: for $x_1 \in H$,

TY $$\begin{aligned} \left\{ {\begin{array}{*{20}l} {z_{n} = J_{{\lambda _{n} }} (x_{n} - \lambda _{n} T^{*} J_{F} (Tx_{n} - Q_{{\mu _{n} }} Tx_{n} )),} \\ {y_{n} = \alpha _{n} x_{n} + (1 - \alpha _{n} )z_{n} ,} \\ {C_{n} = \left\{ {z \in H:\left\| {y_{n} - z} \right\| \le \left\| {x_{n} - z} \right\| } \right\} ,} \\ {D_{n} = \{ z \in H:\langle x_{n} - z,x_{1} - x_{n} \rangle \ge 0\} } \\ {x_{{n + 1}} = P_{{C_{n} \cap D_{n} }} x_{1} .} \\ \end{array} } \right. \end{aligned}$$

They proved the following strong convergence theorem:

### **Theorem TY**

*Let* *H* *be a Hilbert space and let* *F* *be a uniformly convex and smooth Banach space. Let* $J_F$ *be the duality mapping on* *F*. *Let* *A* *and* *B* *be maximal monotone operators of* *H* *into* $2^H$ *and* *F* *into* $2^{F^*}$. *such that* $A^{-1}0 \ne \emptyset$ *and* $B^{-1}0\ne \emptyset$, *respectively. Let* $J_\lambda$ *be the resolvent of* *A* *for* $\lambda >0$ *and let* $Q_\mu$ *be the metric resolvent of* *B* *for* $\mu >0$. *Let* $T : H \rightarrow F$ *be a bounded linear operator such that* $T\ne 0$ *and let* $T^*$ *be the adjoint operator of* *T*. *Suppose that* $A^{-1}0\cap T^{-1}(B^{-1})0 \ne \emptyset$. *Let* $x_1 \in H$ *and let* $\{x_n\}$ *be a sequence generated by (TY), where* $\{\alpha _n\subset [0,1]\}$ *and* $\{\lambda _n\}, \{\mu _n\}$ *satisfy the condition such that*

$$\begin{aligned} 0\le \alpha _n \le \alpha<1, \ \ 0<b\le \mu _n, \ \ 0<c\le r_n\Vert T\Vert ^2\le d<2, \end{aligned}$$

*for some* $a,b,c \in R$. *Then* $\{x_n\}$ *converges strongly to a point* $z_0=P_{A^{-1}0\cap T^{-1}(B^{-1}0)}x_1$.

In this article, a new multidirectional monotone hybrid iteration algorithm for finding a solution to the split common fixed point problem is presented for two countable families of quasi-nonexpansive mappings in Banach spaces. Strong convergence theorems are proved. The application of the result is to consider the split common null point problem of maximal monotone operators in Banach spaces. Strong convergence theorems for finding a solution of the split common null point problem are derived. This iteration algorithm can accelerate the convergence speed of iterative sequence.

Let *E* be a real Banach space with norm $\parallel \cdot \parallel$ and let $E^*$ be the dual space of *E*. We denote the value of $y^* \in E^*$ at $x\in E$ by $\langle x,y^*\rangle$. A Banach space *E* is uniformly convex if for any two sequences $\{x_n\}$ and $\{y_n\}$ in *E* such that

$$\begin{aligned} \lim _{n\rightarrow \infty } \Vert x_n\Vert =\lim _{n\rightarrow \infty } \Vert y_n\Vert =1 \ \ and \ \ \lim _{n\rightarrow \infty }\Vert x_n+y_n\Vert =2, \end{aligned}$$

$\lim _{n\rightarrow \infty }\Vert x_n -y_n\Vert =0$ holds. A uniformly convex Banach space is reflexive.

The duality mapping *J* from *E* into $2^{E^*}$ is defined by

$$\begin{aligned} Jx=\{x^*\in E^* : \langle x,x^*\rangle =\Vert x\Vert ^2=\Vert x^*\Vert ^2\} \end{aligned}$$

for every $x\in E$. Let $U=\{x\in E : \parallel x\parallel =1\}$. The norm of *E* is said to be Gateaux differentiable if for each $x,y\in U$, the limit

$$\begin{aligned} \lim _{t\rightarrow 0}\frac{\parallel x+ty\parallel -\parallel x\parallel }{t} \end{aligned}$$

exists. In the case, *E* is called smooth. We know that *E* is smooth if and only if *J* is a single- valued mapping of *E* into $E^*$. We also know that *E* is reflexive if and only if *J* is surjective, and *E* is strictly convex if and only if *J* is one-to-one. Therefore, if *E* is a smooth, strictly convex and reflexive Banach space, then *J* is a single-valued bijection and in this case, the inverse mapping $J^{-1}$ coincides with the duality mapping $J_*$ on $E^*$. For more details, see Takahashi (2009) and Takahashi (2000).

Let *C* be a nonempty, closed and convex subset of a strictly convex and reflexive Banach space *E*. Then we know that for any $x\in E$, there exists a unique element $z\in C$ such that $\parallel x-z\parallel \le \parallel x-y \parallel$ for all $y\in C$. Putting $z=P_Cx$, we call $P_C$ the metric projection of *E* onto *C*.

### **Definition 1**

Let *E* be a metric space, let $T: D(T)\rightarrow R(T)$ be a mapping with the domain *D*(*T*) and the range *R*(*T*). The mapping *T* is said to be quasi-nonexpansive if

$$\begin{aligned} d(Tx, p)\le d(x,p), \ \forall \ x \in D(T), \ p \in F(T), \end{aligned}$$

where *F*(*T*) is the nonempty fixed point set of *T*.

### **Definition 2**

Let *E* be a smooth Banach space, let $S: D(T)\rightarrow R(T)$ be a mapping with the domain *D*(*T*) and the range *R*(*T*). The mapping *S* is said to be second-type quasi-nonexpansive, if

$$\begin{aligned} \langle Sx-p, J(Sx-x)\rangle \le 0, \ \ \forall \ x \in D(T), \ \ \forall \ p\in F(S), \end{aligned}$$

where *F*(*S*) is the nonempty fixed point set of *T*.

### **Definition 3**

Let *E*, *F* be two normed spaces and *T* be a linear operator from *E* into *F*. The adjoint operator $T^*: F^*\rightarrow E^*$ of *T* is defined by

$$\begin{aligned} f(T(x))=(T^*f)(x), \ \forall \ x \in E, \ f \in F^*, \ \end{aligned}$$

where $E^*$ and $F^*$ are the adjoint spaces of *E* and *F*, respectively.

The adjoint spaces and adjoint operators are very important in the theory of functional analysis and applications. Not only is it an important theoretical subject but it is also a very useful tool in the functional analysis and topological theory.

### **Definition 4**

Let *E* be a Banach space, let *C* be a nonempty, closed, and convex subset of *E*. Let $\{T_n\}$ be sequence of mappings from *C* into itself with nonempty common fixed point set $F=\cap _{n=1}^{\infty }F(T_n)$. The $\{T_n\}$ is said to be uniformly closed if for any convergent sequence $\{z_n\} \subset C$ such that $\Vert T_nz_n-z_n\Vert \rightarrow 0$ as $n\rightarrow \infty$, the limit of $\{z_n\}$ belong to *F*.

## Main results

### **Lemma 5**

*Let* *H* *be a Hilbert space, let* *C* *be a closed convex subset of* *H*, *and let* $\{T_n\}$ *be a uniformly closed family of countable quasi-nonexpansive mappings from* *C* *into itself. Then the common fixed point set* *F* *is closed and convex.*

### *Proof*

Let $p_n \in F$ and $p_n\rightarrow p$ as $n\rightarrow \infty$, we have

$$\begin{aligned} \Vert T_np_n-p_n\Vert \rightarrow 0, \ \ p_n\rightarrow p \end{aligned}$$

as $n\rightarrow \infty$. Since $\{T_n\}$ is uniformly closed, we know that $p \in F$, therefore *F* is closed. Next we show that *F* is also convex. For any $x,y \in F$, let $z=tx+(1-t)y$ for any $t \in (0,1)$, we have

$$\begin{aligned} \Vert T_nz-z\Vert ^2 &= \langle T_nz-z,T_nz-z\rangle \\ &= \Vert T_nz\Vert ^2-2 \langle T_nz,z\rangle + \Vert z\Vert ^2\\ &= \Vert T_nz\Vert ^2-2 \langle T_nz, tx+(1-t)y\rangle + \Vert z\Vert ^2\\ &= \Vert T_nz\Vert ^2-2 t\langle T_nz, x \rangle -2(1-t)\langle T_nz, y\rangle + \Vert z\Vert ^2\\ &= t\Vert T_nz\Vert ^2+(1-t)\Vert T_nz\Vert ^2+t\Vert x\Vert ^2-t\Vert x\Vert ^2+(1-t)\Vert y\Vert ^2\\ &\quad -(1-t)\Vert y\Vert ^2-2 t\langle T_nz, x \rangle -2(1-t)\langle T_nz, y\rangle + \Vert z\Vert ^2\\ &=t(\Vert T_nz\Vert ^2-2 t\langle T_nz, x \rangle +\Vert x\Vert ^2)+(1-t)(\Vert T_nz\Vert ^2-2 t\langle T_nz, y \rangle +\Vert y\Vert ^2)\\ &\quad -t\Vert x\Vert ^2-(1-t)\Vert y\Vert ^2+\Vert z\Vert ^2\\ &=t\langle T_nz-x, T_nz-x \rangle +(1-t)\langle T_nz-y, T_nz-y \rangle \\ &\quad -t\Vert x\Vert ^2-(1-t)\Vert y\Vert ^2+\Vert z\Vert ^2\\ &=t \Vert T_nz-x\Vert ^2+(1-t)\Vert T_nz-y\Vert ^2-t\Vert x\Vert ^2-(1-t)\Vert y\Vert ^2+\Vert z\Vert ^2\\ &\le t \Vert z-x\Vert ^2+(1-t)\Vert z-y\Vert ^2-t\Vert x\Vert ^2-(1-t)\Vert y\Vert ^2+\Vert z\Vert ^2\\ &= \Vert z\Vert ^2- 2\langle z, z\rangle +\Vert z\Vert ^2=0, \end{aligned}$$

for all *n*. This implies $z \in F$, therefore *F* is convex. This completes the proof. $\square$

### **Lemma 6**

*Let* *E* *be a smooth Banach space, let* *C* *be a closed convex subset of* *E*, *and let* $\{S_n\}$ *be a uniformly closed family of countable second-type quasi-nonexpansive mappings from* *C* *into itself. Then the common fixed point set* *F* *is closed and convex.*

### *Proof*

Let $p_n \in F$ and $p_n\rightarrow p$ as $n\rightarrow \infty$, we have

$$\begin{aligned} \Vert T_np_n-p_n\Vert \rightarrow 0, \quad p_n\rightarrow p \end{aligned}$$

as $n\rightarrow \infty$. Since $\{T_n\}$ is uniformly closed, we know that $p \in F$, therefore *F* is closed. Next we show that *F* is also convex. For any $x,y \in F$, let $z=tx+(1-t)y$ for any $t \in (0,1)$, we have

$$\begin{aligned} \langle S_nz-x,J(S_nz-z)\rangle \le 0, \\ \langle S_nz-y,\quad J(S_nz-z)\rangle \le 0. \end{aligned}$$

From the two inequalities given above, we have that

$$\begin{aligned} \langle t(S_nz-x)+(1-t)(S_nz-y),J(S_nz-z)\rangle \le 0 \end{aligned}$$

which implies

$$\begin{aligned} \langle S_nz-z,J(S_nz-z)\rangle \le 0. \end{aligned}$$

Therefore $\Vert S_nz-z\Vert ^2\le 0$, that is $\Vert S_nz-z\Vert ^2= 0$, so that $z\in F.$ Therefore *F* is convex. This completes the proof. $\square$

### **Lemma 7**

(Alber 1996) *Let* *H* *be a Hilbert space, let* *C* *be a nonempty closed convex subset of* *H* *and let* $x \in E.$ *Then*

$$\begin{aligned} \Vert z-P_Cx\Vert ^2+\Vert P_Cx-x\Vert ^2\le \Vert z-x\Vert ^2, \quad \forall \ z \in C. \end{aligned}$$

Next, we present a new hybrid algorithm so-called the multidirectional hybrid algorithm for finding the common fixed point of a uniformly closed family of countable quasi-nonexpansive mappings and a uniformly closed family of countable second-type quasi-nonexpansive mappings.

### **Theorem 8**

*Let* *H* *be a Hilbert space and let* *E* *be a uniformly convex and smooth Banach space. Let* *J* *be the duality mapping on* *E*. *Let* $\{T_n\}: H\rightarrow H$ *be a uniformly closed family of countable quasi-nonexpansive mappings with the nonempty common fixed point set* $\cap _{n=1}^{\infty }F(T_n)$ *and* $\{S_n\}: E\rightarrow E$ *be a uniformly closed family of countable second-type quasi-nonexpansive mappings with the nonempty common fixed point sets* $\cap _{n=1}^{\infty }F(S_n)$. *Suppose that* $F=(\cap _{n=1}^{\infty }F(T_n))\cap (T^{-1}(\cap _{n=1}^{\infty }F(S_n)))\ne \emptyset$. *Let* $T : H \rightarrow E$ *be a bounded linear operator such that* $T\ne 0$ *and let* $T^*$ *be the adjoint operator of* *T*. *Let* $x_{1,i}\in H, i=1,2,3, \ldots ,N$ *and let* $\{x_n\}$ *and* $\{z_n\}$ *be two sequences generated by*

$$\begin{aligned} {\left\{ \begin{array}{ll} z_n=T_{n}(x_n-r_nT^*J(Tx_n-S_{n}Tx_n)),\\ C_n=\{z\in C_{n-1} : \Vert z_n-z\Vert \le \Vert x_n-z\Vert \},\\ C_0=H,\ n=1,2, 3, \ldots , \\ x_{n+1}=\sum \nolimits _{i=1}^{N}\lambda _i P_{C_n}x_{1,i}, \ \sum \nolimits _{i=1}^{N}\lambda _i= 1,\\ \end{array}\right. } \end{aligned}$$

*where* $\{r_n\}$ *satisfy the condition such that*

$$\begin{aligned} 0<a\le r_n\Vert T\Vert ^2\le b<2, \end{aligned}$$

*for some constants* *a*, *b* *and* $\lambda \in [0,1]$ *is a constant. Then the following conclusions hold:*

(1) $\{x_n\}$ and $\{z_n\}$ *converge strongly to a point* $w \in F$;

(2) *the limits* $$\lim _{n\rightarrow \infty }P_{C_n}x_{1,i}=P_{F}x_{1,i}, \ i=1,2,3, \ldots ,N$$;

(3) $$w=\sum _{i=1}^{N}\lambda _i \lim _{n\rightarrow \infty } P_{C_n}x_{1,i}$$.

### *Proof*

It is not hard to see that, $C_n$ is closed and convex for all $n\ge 0$. Let us show that, $F\subset C_n$ for all $n\ge 0$. For any $z \in F$, we have

2 $$\begin{aligned} \begin{aligned} \Vert z_n-z\Vert ^2&=\Vert T_{n}(x_n-r_nT^*J(Tx_n-S_{n}Tx_n))-z\Vert ^2\\&\le \Vert x_n-r_nT^*J(Tx_n-S_{n}Tx_n))-z\Vert ^2\\&=\Vert x_n-z\Vert ^2-2\langle x_n-z, r_nT^*J(Tx_n-S_{n}Tx_n)\rangle \\&\quad +\Vert r_nT^*J(Tx_n-S_{n}Tx_n))\Vert ^2\\&\le \Vert x_n-z\Vert ^2-2r_n\langle Tx_n-Tz, J(Tx_n-S_{n}Tx_n)\rangle \\&\quad +r_n^2 \Vert T\Vert ^2\Vert J(Tx_n-S_{n}Tx_n))\Vert ^2\\&= \Vert x_n-z\Vert ^2+r_n^2 \Vert T\Vert ^2\Vert Tx_n-S_{n}Tx_n)\Vert ^2 \\&\quad -2r_n\langle Tx_n-S_{n}Tx_n+S_{n}Tx_n-Tz, J(Tx_n-S_{n}Tx_n)\rangle \\&= \Vert x_n-z\Vert ^2+r_n^2 \Vert T\Vert ^2\Vert Tx_n-S_{n}Tx_n)\Vert ^2 \\&\quad -2r_n\langle S_{n}Tx_n-Tz, J(Tx_n-S_{n}Tx_n)\rangle -2r_n \Vert Tx_n-S_{n}Tx_n)\Vert ^2\\&= \Vert x_n-z\Vert ^2+r_n^2 \Vert T\Vert ^2\Vert Tx_n-S_{n}Tx_n)\Vert ^2 -2r_n \Vert Tx_n-S_{n}Tx_n)\Vert ^2\\&= \Vert x_n-z\Vert ^2+r_n(r_n\Vert T\Vert ^2-2)\Vert Tx_n-S_{n}Tx_n)\Vert ^2\\&\le \Vert x_n-z\Vert ^2. \end{aligned} \end{aligned}$$

So, $z\in C_n$ , which implies that $F\subset C_n$ for all $n\ge 0$.

Let $u_{n+1,i}=P_{C_n}x_{1,i}$ for all $n\ge 1, \ i=1,2,3, \ldots ,N$. Since *F* is nonempty, closed, and convex, there exist $p_{1,i}=P_{F}x_{1,i}$ such that

$$\begin{aligned} \Vert u_{n+1,i}-p_{1,i}\Vert \le \Vert x_{1,i}-p_{1,i}\Vert , \quad i=1,2,3, \ldots ,N. \end{aligned}$$

This means that $\{u_{n,i}\}$ is bounded for all $i=1,2,3, \ldots ,N$.

From $u_{n+1,i}=P_{C_n}x_{1,i}$ and $C_{n}\subset C_{n-1}$, we have that

$$\begin{aligned} \Vert u_{n,i}-x_{1,i}\Vert \le \Vert u_{n+1,i}-x_{1,i}\Vert , \quad i=1,2,3, \ldots ,N, \end{aligned}$$

for all $n\in N$. This implies that $\{\Vert u_{n,i}-x_1\Vert \}$ is bounded and nondecreasing for all $i=1,2,3, \ldots ,N$. Then there exist the limits of $\{\Vert u_{n,i}-x_{1,i}\Vert : i=1,2,3, \ldots ,N\}$. Put

$$\begin{aligned} \lim _{n\rightarrow \infty }\Vert u_{n,i}-x_{1,i}\parallel =c_i, \quad i=1,2,3, \ldots ,N. \end{aligned}$$

On the other hand, $u_{n+m,i}\in C_{n-1},\ i=1,2,3, \ldots ,N$, by using Lemma 7, we have, for any positive integer *m*, that

$$\begin{aligned} \Vert u_{n+m,i}-u_{n,i}\Vert ^2\le \Vert u_{n+m,i}-x_{1,i}\Vert ^2-\Vert u_{n,i}-x_{1,i}\Vert ^2. \end{aligned}$$

So that $\{u_{n,i}\}$ is Cauchy sequences in *C* for all $i=1,2,3, \ldots ,N$, therefore there exit two points $p_i\in C$ such that

$$\begin{aligned} \lim _{n\rightarrow \infty }u_{n,i}=p_i, \quad i=1,2,3, \ldots ,N. \end{aligned}$$

That is

$$\begin{aligned} \lim _{n\rightarrow \infty }P_{C_n}x_{1,i}=p_i, \quad i=1,2,3, \ldots ,N. \end{aligned}$$

Therefore

$$\begin{aligned} \lim _{n\rightarrow \infty }x_n=\sum _{i=1}^{N} \lambda _i p_i. \end{aligned}$$

Since $x_{n+1} \in C_n$, we have

$$\begin{aligned} \Vert z_n-x_{n+1}\Vert \le \Vert x_n-x_{n+1}\Vert \end{aligned}$$

which implies

$$\begin{aligned} \lim _{n\rightarrow \infty }z_n=\sum _{i=1}^{N} \lambda _i p_i. \end{aligned}$$

From (2), we have, for any $z\in F$, that

$$\begin{aligned} r_n(2-r_n\Vert T\Vert ^2)\Vert Tx_n-Q_{t_n}Tx_n)\Vert ^2\le \Vert x_n-z\Vert ^2-\Vert z_n-z\Vert ^2\rightarrow 0 \end{aligned}$$

as $n\rightarrow \infty$. This implies

3 $$\begin{aligned} \lim _{n\rightarrow \infty }\Vert Tx_n-S_{n}Tx_n)\Vert =0. \end{aligned}$$

Since

$$\begin{aligned} \lim _{n\rightarrow \infty }Tx_n=T\left( \sum _{i=1}^{N} \lambda _i p_i\right) \end{aligned}$$

and the sequence $\{S_{n}\}$ is uniformly closed, so that

$$\begin{aligned} T\left( \sum _{i=1}^{N} \lambda _i p_i\right) \in \cap _{n=1}^{\infty }F(S_{n}). \end{aligned}$$

That is

$$\begin{aligned} \sum _{i=1}^{N} \lambda _i p_i \in T^{-1}( \cap _{n=1}^{\infty }F(S_{n})). \end{aligned}$$

On the other hand, from

$$\begin{aligned} z_n=T_{n}(x_n-r_nT^*J(Tx_n-S_{n}Tx_n)), \end{aligned}$$

we have

$$\begin{aligned} \Vert z_n-T_{n}z_n\Vert&=\Vert T_{n}(x_n-r_nT^*J(Tx_n-S_{n}Tx_n))-T_{n}z_n\Vert \\&\le \Vert (x_n-r_nT^*J(Tx_n-S_{n}Tx_n))-z_n\Vert . \end{aligned}$$

This together with (3) implies that

$$\begin{aligned} \lim _{n\rightarrow \infty }\Vert z_n-T_{n}z_n\Vert =0. \end{aligned}$$

Since

$$\begin{aligned} \lim _{n\rightarrow \infty }z_n=\sum _{i=1}^{N} \lambda _i p_i, \end{aligned}$$

and the sequence $\{T_n\}$ is uniformly closed, so that

$$\begin{aligned} \sum _{i=1}^{N} \lambda _i p_i\in \cap _{n=1}^{\infty }F(T_{n}). \end{aligned}$$

From above two hands, we have $\sum _{i=1}^{N} \lambda _i p_i\in F$.

Finally, we prove $p_i=P_{F}x_{1,i}, \ i=1,2,3, \ldots ,N$. From Lemma 7, we have

4 $$\begin{aligned} \Vert p_i- P_{F}x_{1,i}\Vert ^2+\Vert P_{F}x_{1,i}- x_{1,i}\Vert ^2\le \Vert p_i- x_{1,i}\Vert ^2. \end{aligned}$$

On the other hand, since $x_{n+1,i}= P_{C_n}x_{1,i}$ and $F \subset C_n$, for all *n*. Also from Lemma 7, we have

5 $$\begin{aligned} \Vert P_{F}x_{1,i}- x_{n+1,i}\Vert ^2+\Vert x_{n+1,i}- x_{1,i}\Vert ^2\le \Vert P_{F}x_{1,i}- x_{1,i}\Vert ^2. \end{aligned}$$

Since

6 $$\begin{aligned} \lim _{n\rightarrow \infty }\Vert x_{n+1,i}- x_{1,i}\Vert =\Vert p_i-x_{1,i}\Vert . \end{aligned}$$

Combining (4), (5) and (6), we know that $\Vert p_i-x_{1,i}\Vert =\Vert P_{F}x_{1,i}- x_{1,i}\Vert$. Therefore, it follows from the uniqueness of $P_{F}x_{1,i}$ that $p_i= P_{F}x_{1,i}$. This completes the proof. $\square$

By using Theorem  8 and setting $N = 1$, we can get the following result.

### **Theorem 9**

*Let* *H* *be a Hilbert space and let* *E* *be a uniformly convex and smooth Banach space. Let* *J* *be the duality mapping on* *E*. *Let* $\{T_n\}: H\rightarrow H$ *be a uniformly closed family of countable quasi-nonexpansive mappings with the nonempty common fixed point set* $\cap _{n=1}^{\infty }F(T_n)$ *and* $\{S_n\}: E\rightarrow E$ *be a uniformly closed family of countable second-type quasi-nonexpansive mappings with the nonempty common fixed point sets* $\cap _{n=1}^{\infty }F(S_n)$. *Suppose that* $F=(\cap _{n=1}^{\infty }F(T_n))\cap (T^{-1}(\cap _{n=1}^{\infty }F(S_n)))\ne \emptyset$. *Let* $T : H \rightarrow E$ *be a bounded linear operator such that* $T\ne 0$ *and let* $T^*$ *be the adjoint operator of* *T*. *Let* $x_1\in H$ *and let* $\{x_n\}$ *be a sequence generated by*

$$\begin{aligned} {\left\{ \begin{array}{ll} z_n=T_{n}(x_n-r_nT^*J(Tx_n-S_{n}Tx_n)),\\ C_n=\{z\in C_{n-1} : \Vert z_n-z\Vert \le \Vert x_n-z\Vert \},\\ C_0=H,\ n=1,2, 3, \ldots , \\ x_{n+1}=P_{C_n}x_1 , \end{array}\right. } \end{aligned}$$

*where* $\{r_n\}$ *satisfy the condition such that*

$$\begin{aligned} 0<a\le r_n\Vert T\Vert ^2\le b<2, \end{aligned}$$

*for some constants* *a*, *b*. *Then* $\{x_n\}$ *converges strongly to a point* $z_0=P_{F}x_1$.

## Application for common null point problem

Let *E* be a Banach space, let *A* be a multi-valued operator from *E* to $E^*$ with domain $D(A)=\{z\in E: Az\ne \emptyset \}$ and range $R(A)=\{z\in E: z\in D(A)\}$. An operator A is said to be monotone if

$$\begin{aligned} \langle x_1-x_2, y_1-y_2 \rangle \ge 0 \end{aligned}$$

for each $x_1, x_2 \in D(A)$ and $y_1\in Ax_1, y_2\in Ax_2$. A monotone operator A is said to be maximal if it’s graph $G(A)=\{(x, y) : y \in Ax\}$ is not properly contained in the graph of any other monotone operator. We know that if *A* is a maximal monotone operator, then $A^{-1}0$ is closed and convex. The following result is also well-known.

### **Theorem 10**

(Rockafellar 1970). *Let* *E* *be a reflexive, strictly convex and smooth Banach space and let* *A* *be a monotone operator from* *E* *to* $E^{*}$. *Then* *A* *is maximal if and only if* $R(J + rA)=E^*$. *for all* $r>0$.

Let *E* be a reflexive, strictly convex and smooth Banach space, and let *A* be a maximal monotone operator from *E* to $E^*$. Using Theorem  10 and strict convexity of *E*, we obtain that for every $r>0$ and $x\in E$, there exists a unique $x_r$ such that

$$\begin{aligned} Jx\in Jx_r+rAx_r. \end{aligned}$$

Then we can define a single valued mapping $J_r :E \rightarrow D(A)$ by $J_r = (J + rA)^{-1}J$ and such a $J_r$ is called the resolvent of *A*. We know that $J_r$ is a nonexpansive mapping and $A^{-1}0=F(J_r)$ for all $r > 0$, see Takahashi (2000, 2009), Alber (1996).

### **Lemma 11**

(Aoyama et al. 2009) *Let* *E* *be a reflexive, strictly convex and smooth Banach space, and let* *A* *be a maximal monotone operator from* *E* *to* $E^*$. *Then*

$$\begin{aligned} \langle J_rx-p, J(x-J_rx)\rangle \ge 0, \quad \forall \ x \in E, \quad \forall \quad p \in A^{-1}0, \quad \forall \ r>0, \end{aligned}$$

*where* $J_{r}$ *is the resolvent of* *A*.

From Lemma 11, we know that, $J_r$ is a second-type quasi-nonexpansive mapping, where $J_r$ is the resolvent of *A* with $r>0.$

### **Definition 12**

Let *E* be a Banach space, let *C* be a nonempty, closed, and convex subset of *E*. Let $\{T_n\}$ be sequence of mappings from *C* into itself with nonempty common fixed point set $F=\cap _{n=1}^{\infty }F(T_n)$. The $\{T_n\}$ is said to be uniformly weak closed if for any weak convergent sequence $\{z_n\} \subset C$ such that $\Vert T_nz_n-z_n\Vert \rightarrow 0$ as $n\rightarrow \infty$, the weak limit of $\{z_n\}$ belong to *F*.

A uniformly weak closed family of countable quasi-nonexpansive mappings must be a uniformly closed family of countable quasi-nonexpansive mappings.

### **Theorem 13**

*Let* $r_n\ge c >0$, *for some constant* *c*, *then* $\{J^A_{r_n}\}_{n=0}^{\infty }$ *is a uniformly weak closed family of countable quasi-nonexpansive mappings with the nonempty common fixed point sets* $\bigcap _{n=0}^{\infty }F(J^A_{r_n})=A^{-1}0$.

### *Proof*

It is well-known that, $\bigcap _{n=0}^{\infty }F(J^A_{r_n})=A^{-1}0 \ne \emptyset$ and $\{J^A_{r_n}\}_{n=0}^{\infty }$ is a family of countable nonexpansive mappings. Let $\{z_n\}\subset E$ be a sequence such that $z_n \rightharpoonup p$ and $\lim _{n\rightarrow \infty }\Vert z_n-J^A_{r_n}z_n\Vert =0$. Since *J* is uniformly norm-to-norm continuous on bounded sets, we obtain

$$\begin{aligned} \frac{1}{r_n}(Jz_n-JJ^A_{r_n}z_n)\rightarrow 0. \end{aligned}$$

It follows from

$$\begin{aligned} \frac{1}{r_n}(Jz_n-JJ^A_{r_n}z_n)\in AJ^A_{r_n}z_n \end{aligned}$$

and the monotonicity of *A* that

$$\begin{aligned} \left\langle w-J^A_{r_n}z_n, w^*-\frac{1}{r_n}(Jz_n-JJ^A_{r_n}z_n)\right\rangle \ge 0 \end{aligned}$$

for all $w\in D(A)$ and $w^*\in Aw$. Letting $n\rightarrow \infty$, we have $\langle w-p, w^*\rangle \ge 0$ for all $w\in D(A)$ and $w^*\in Aw$. Therefore from the maximality of *A*, we obtain $p\in A^{-1}0$. That is $p\in \bigcap _{n=0}^{\infty }F(J^A_{r_n})$. This completes the proof. $\square$

### **Theorem 14**

*Let* *H* *be a Hilbert space and let* *F* *be a uniformly convex and smooth Banach space. Let* $J_F$ *be the duality mapping on* *F*. *Let* *A* *and* *B* *be maximal monotone operators of* *H* *into* $2^H$ *and* *F* *into* $2^F$ *such that* $A^{-1}0\ne \emptyset$ *and* $B^{-1}0\ne \emptyset$ , *respectively. Let* $J_r$ *be the resolvent of* *A* *for* $r>0$ *and let* $Q_\mu$ *be the metric resolvent of B for* $\mu >0$. *Let* $T : H \rightarrow F$ *be a bounded linear operator such that* $T\ne 0$ *and let* $T^*$ *be the adjoint operator of* *T*. *Suppose that* $W=A^{-1}0\cap T^{-1}(B^{-1}0)\ne \emptyset$. *Let* $x_1\in H$ *and let* $\{x_n\}$ *be a sequence generated by*

$$\begin{aligned} {\left\{ \begin{array}{ll} z_n=J_{r_n}(x_n-r_nT^*J_F(Tx_n-Q_{t_n}Tx_n)),\\ C_n=\{z\in C_{n-1} : \Vert z_n-z\Vert \le \Vert x_n-z\Vert \},\\ C_0=H,\ n=1,2, 3, \ldots , \\ x_{n+1}=P_{C_n}x_1 ,\end{array}\right. } \end{aligned}$$

*where* $\{r_n\}$ *satisfy the condition such that*

$$\begin{aligned} 0<a\le r_n\Vert T\Vert ^2\le b<2,\quad 0<c\le t_n, \end{aligned}$$

*for some constants* *a*, *b*, *c*. *Then* $\{x_n\}$ *converges strongly to a point* $z_0=P_{W}x_1$.

### Proof

Let $T_n=J_{r_n}, \ S_n=Q_{\mu _n}$ for all $n\ge 1$, then $\{T_n\}, \ \{S_n\}$ satisfy the all conditions of Theorem  8, and

$$\begin{aligned} F=\cap _{n=1}^{\infty }F(T_n)\cap T^{-1}(\cap _{n=1}^{\infty }F(S_n))= A^{-1}0\cap T^{-1}(B^{-1}0)=W. \end{aligned}$$

By using Theorem  9, we obtain the conclusion of Theorem  14. This completes the proof. $\square$

### **Theorem 15**

*Let* *H* *be a Hilbert space and let* *F* *be a uniformly convex and smooth Banach space. Let* $J_F$ *be the duality mapping on* *F*. *Let* *A* *and* *B* *be maximal monotone operators of* *H* *into* $2^H$ *and* *F* *into* $2^F$ *such that* $A^{-1}0\ne \emptyset$ *and* $B^{-1}0\ne \emptyset$ , *respectively. Let* $J_r$ *be the resolvent of* *A* *for* $r>0$ *and let* $Q_\mu$ *be the metric resolvent of B for* $\mu >0$. *Let* $T : H \rightarrow F$ *be a bounded linear operator such that* $T\ne 0$ *and let* $T^*$ *be the adjoint operator of* *T*. *Suppose that* $W=A^{-1}0\cap T^{-1}(B^{-1}0)\ne \emptyset$. *Let* $x_{1,i}\in H$ *and let* $\{x_n\}$ *and* $\{z_n\}$ *be two sequences generated by*

$$\begin{aligned}{\left\{ \begin{array}{ll} z_n=J_{r_n}(x_n-r_nT^*J_F(Tx_n-Q_{t_n}Tx_n)),\\ C_n=\{z\in C_{n-1} : \Vert z_n-z\Vert \le \Vert x_n-z\Vert \},\\ C_0=H,\ n=1,2, 3, \ldots , \\ x_{n+1}=\sum _{i=1}^{N}\lambda _i P_{C_n}x_{1,i}, \, \sum _{i=1}^{N}\lambda _i= 1, \end{array}\right. } \end{aligned}$$

*where* $\{r_n\}$ *satisfy the condition such that*

$$\begin{aligned} 0<a\le r_n\Vert T\Vert ^2\le b<2, \end{aligned}$$

*for some constants* *a*, *b* *and* $\lambda \in [0,1]$ *is a constant. Then the following conclusions hold:*

(1) $\{x_n\}$ and $\{z_n\}$ converge strongly to a point $w \in W$;

(2) the limits $$\lim _{n\rightarrow \infty }P_{C_n}x_{1,i}=P_{W}x_{1,i}, \ i=1,2,3, \ldots ,N$$;

(3) $$w=\sum _{i=1}^{N}\lambda _i \lim _{n\rightarrow \infty } P_{C_n}x_{1,i}$$.

### *Proof*

Let $T_n=J_{r_n}, \ S_n=Q_{\mu _n}$ for all $n\ge 1$, then $\{T_n\}, \ \{S_n\}$ satisfy the all conditions of Theorem  9, and

$$\begin{aligned} F=\cap _{n=1}^{\infty }F(T_n)\cap T^{-1}(\cap _{n=1}^{\infty }F(S_n))= A^{-1}0\cap T^{-1}(B^{-1}0)=W. \end{aligned}$$

By using Theorem  8, we obtain the conclusion of Theorem  15. This completes the proof. $\square$

## Examples

It is easy to see that, a uniformly weak closed family $\{T_n\}$ of countable quasi-nonexpansive mappings must be a uniformly closed family $\{T_n\}$ of countable quasi-nonexpansive mappings. Next we will give an example which is a uniformly closed family of countable quasi-nonexpansive mappings, but not a uniformly weak closed family of countable quasi-nonexpansive mappings.

### **Conclusion 16**

*Let* *H* *be a Hilbert space,* $\{x_n\}_{n=1}^{\infty }\subset H$ *be a sequence such that it converges weakly to a non-zero element* $x_0$ *and* $\Vert x_i-x_j\Vert \ge 1$ *for any* $i\ne j$. *Define a sequence of mappings* $T_n: H\rightarrow H$ *as follows*

$$\begin{aligned}T_n(x)=\left\{ \begin{array}{ll} L_nx_n\ &{} if \quad x=x_n (\exists \ n\ge 1) ,\\ -x &{} if \quad x\ne x_n (\forall \ n\ge 1), \end{array}\right. \end{aligned}$$

*where* $L_n\le 1$ *and* $\lim _{n\rightarrow \infty }L_n=1$. *Then* $\{T_n\}$ *is a uniformly closed family of countable quasi-nonexpansive mappings with the common fixed point set* $F=\{0\}$, *but not a uniformly weak closed family of countable quasi-nonexpansive mappings.*

### Proof

It is obvious that, $\{T_n\}$ has a unique common fixed point 0. Next, we prove that, $\{T_n\}$ is uniformly closed. In fact that, for any strong convergent sequence $\{z_n\}\subset E$ such that $z_n\rightarrow z_0$ and $\Vert z_n-T_nz_n\Vert \rightarrow 0$ as $n\rightarrow \infty$, there exists sufficiently large nature number *N* such that $z_n\ne x_m$, for any $n, m >N$. Then $T_nz_n=-z_n$ for $n>N$, it follows from $\Vert z_n-T_nz_n\Vert \rightarrow 0$ that $2z_n\rightarrow 0$ and hence $z_0 \in F$. From the definition of $\{T_n\}$, we have

$$\begin{aligned} \Vert T_nx-0\Vert = \Vert T_nx\Vert \le \Vert L_nx\Vert =\Vert x-0\Vert , \quad \forall \, x \in H. \end{aligned}$$

so that $\{T_n\}$ is a uniformly closed family of countable quasi-nonexpansive mappings. Next, we prove the $\{T_n\}$ is not weak closed. Since $\{x_n\}$ converges weakly to $x_0$ and

$$\begin{aligned} \Vert T_nx_n-x_n\Vert =\Vert L_nx_n-x_n\Vert =(L_n-1)\Vert x_n\Vert \rightarrow 0 \end{aligned}$$

as $n\rightarrow \infty$, but $x_0$ is not a fixed point.$\Box$

## Conclusion

In the multidirectional iteration algorithm, the $C_n$ is a closed convex set, and $F\subset C_n$ for any $n\ge 1$. If we use one initial $x_{1,1}$, the projection point $x_n=P_{C_n}x_{1,1}$ belongs to the boundary of the $C_n$. If we use *N* initials $x_{1,1}, x_{1,2}, x_{1,3}, \ldots , x_{1,N}$, the element $x_{n}=\sum _{i=1}^{N}\lambda _i P_{C_n}x_{1,i}$ belongs to the interior of the $C_n$. In general, the distance $d(\sum _{i=1}^{N}\lambda _i P_{C_n}x_{1,i}, F)$ is less than the distance $d(P_{C_n}x_{1,1}, F)$, so the multidirectional iteration algorithm can accelerate the convergence speed of iterative sequence $\{x_n\}$. We give a simple experimental example in the following.

### Example

Let $X=R^2, \ C_n=\{(x,y)\in R^2: x^2+y^2\le 1\}, \ x_{1,1}=(1,1), \ x_{1,2}=(-1,1), \ F=\{0\}$. Case 1, take only one initial $x_{1,1}$, $x_n=P_{C_n}x_{1,1}=(\frac{\sqrt{2}}{2},\frac{\sqrt{2}}{2})$, then $d(x_n, F)=1$. Case 2, take two initials $x_{1,1},x_{1.2}$,

$$\begin{aligned} x_n=\frac{1}{2}P_{C_n}x_{1,1}+\frac{1}{2}P_{C_n}x_{1,2} =\left( 0,\frac{\sqrt{2}}{2}\right) , \end{aligned}$$

then $d(x_n, F)=\frac{\sqrt{2}}{2}$. From the inequality “$\frac{\sqrt{2}}{2}< 1$”, we can see that, the multidirectional iteration algorithm can accelerate the convergence speed of iterative sequence $\{x_n\}$.
